# Controlled Reassociation of Multistranded, Polycrossover DNA Molecules into Double Helices

**DOI:** 10.1021/acs.nanolett.5c04286

**Published:** 2025-11-03

**Authors:** Nada Kabbara, Lauren A. Anderson, Shubhajit Singha, Arun Richard Chandrasekaran

**Affiliations:** † Department of Nanoscale Science and Engineering, 1084 University at Albany, State University of New York, Albany, New York 12222, United States; ‡ Department of Chemistry, 1084 University at Albany, State University of New York, Albany, New York 12222, United States; § The RNA Institute, 1084 University at Albany, State University of New York, Albany, New York 12222, United States

**Keywords:** DNA nanotechnology, DNA devices, paranemic crossover DNA, strand displacement, DNA nanostructures

## Abstract

Shape-changing DNA nanostructures have found applications in biosensing, drug delivery, and data storage. Here, we use sequence and temperature-controlled reassociation of one type of a DNA nanostructure (paranemic crossover (PX) DNA) into another structure (duplex). In the presence of an anti-PX structure that is composed of strands that are each complementary to those in PX DNA, the structures reassociate at specific temperatures to form duplexes. Using the denaturing agent formamide, we decreased the temperature required for this reassociation. We demonstrate tunable biostability, where the structures before and after reassociation show vastly different nuclease resistance against DNase I. We further extend the strategy to other polycrossover DNA molecules such as a double crossover motif and a juxtaposed DNA motif, showing controlled reassociation of different DNA motifs into duplexes. Our study highlights the potential for DNA motifs to function as switchable molecular systems, offering new insights for DNA-based materials and devices.

## Graphical abstract

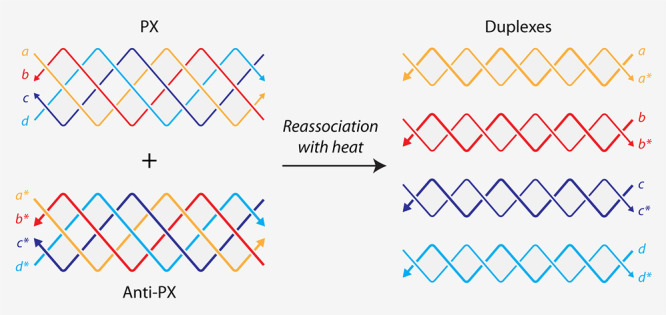

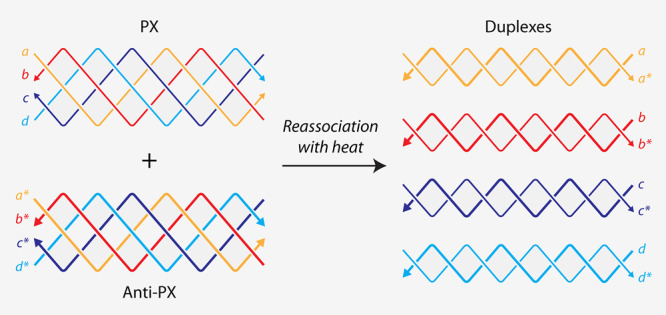

Reconfigurable DNA nanostructures play a key role in applying dynamic DNA nanotechnology in fields such as diagnostics, drug delivery, molecular computation and reactive circuits.^1^ Typically, reconfiguration is achieved through environmental (temperature^2^), physical (light^3^), chemical (pH^4^) and biomolecular (DNA,^5^ proteins,^6^ antibodies^7^) stimuli. For biomolecular reactions, while toehold-based DNA strand displacement^8^ is an often-used strategy to reconfigure DNA nanostructures, several recent works have focused on creating toehold-less strand displacement using weaker DNA interactions,^9^ enzymes,^10,11^ or sequence composition.^12^ While sequence-based affinity is widely used in strand displacement reactions, we recently showed structure-based affinity as an alternate strategy to reconfigure one DNA structure into another.^13^ In some cases, sequence-defined control over DNA nanostructure reconfiguration can be additionally controlled by environmental stimuli, resulting in structures with largely different properties.

In addition to developments in reconfigurable DNA nanostructures, there has been considerable effort toward the minimalistic synthesis of DNA nanostructures and simpler assembly routes.^14^ Only one component strand can be designed to self-assemble into nanotubes,^15^ 1D and 2D arrays,^16^ and 3D crystals,^17^ while two component strands can assemble into size-defined nanoprisms.^18^ Similarly, two individual strands of a DNA duplex can each be folded into DNA nanostructures, indicating the ability to create structures (eg: triangles) that are different from the starting material (e.g., a duplex).^19^ Enzymatic digestion of component DNA or RNA strands in duplexes can also trigger the formation of DNA or RNA cubes from the undigested strands.^20^ While component strands of a duplex can reassemble into DNA nanostructures, the opposite strategy has also been developed, where DNA nanostructures can reassociate into duplexes. The Afonin group has led the development of this strategy, constructing DNA cubes,^21^ RNA cubes,^21^ and RNA polygons^22^ that can interact with other nucleic acid cubes or polygons respectively to form duplexes. Reassociation of these structures into duplexes allowed additional functionality, such as combining two split aptamers into a reactive aptamer or creating active siRNA molecules from inactive starting materials.^23–25^ In these developments, the DNA and RNA nanostructures were made of double helical edges, and the structures reassociate into double helices.

In this work, we explored whether multistranded DNA motifs that contain multiple crossovers can reassociate into DNA duplexes. We present evidence for reassociation of a model nanostructure, the paranemic crossover (PX) DNA motif,^26^ into duplexes, with strategies to reduce the temperature requirement for the reassociation through the use of a denaturing agent. PX DNA contains two adjacent double helical domains connected by six crossovers, and has been used in the creation of 1D^27^ and 2D arrays,^28^ in the cohesion of topologically closed molecules,^29^ and in creation of single-stranded knots^30^ and origami.^31^ Furthermore, the PX structure has been implied to have biological roles in homologous recombination,^32^ making it a good polycrossover model structure for demonstrating this strategy. We also show that reassociation is possible in other polycrossover DNA motifs with different number of crossovers, with their thermal stability dictating the reassociation temperatures.

To evaluate whether PX molecules can reassociate into duplexes, we chose a PX sequence we have used in our earlier study (Figure S1).^33^ We designed an anti-PX structure that is assembled from reverse complements of each of the four strands in the PX molecule (Figure 1 and Figure S1). That is, each strand in the PX (indicated by “a”, “b”, “c”, and “d” in Figure 1) has a fully complementary partner in the anti-PX (designated as “a*”, “b*”, “c*”, and “d*”, respectively). We set out to find whether the component strands of the PX and anti-PX molecules reassociate to form duplexes. We first validated assembly of the PX and anti-PX complexes in tris-acetate-EDTA (TAE) buffer containing 12.5 mM Mg^2+^ using nondenaturing polyacrylamide gel electrophoresis (Figure 2a). Both structures formed with near-efficient assembly yields. We also assembled the four individual duplexes and validated them on a nondenaturing gel. The PX and anti-PX showed melting temperatures of 59.4 and 61 °C respectively, consistent with earlier reports,^34^ while the melting temperature of the duplexes ranged between ∼74 and 81 °C (Figure 2b and Figure S2).

**Figure 1:**
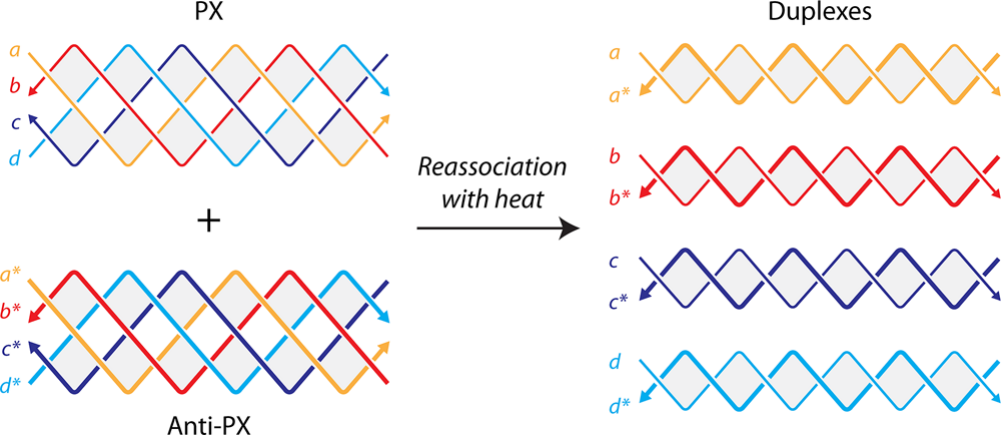
Reassociation of PX and anti-PX molecules into duplexes. The PX structure contains four strands hybridized into two adjacent double helical domains connected by six crossovers. Sequences of the strands in the PX are indicated by the letters a, b, c and d, each of which are 38 nucleotides long. The component strands of anti-PX molecule a*, b*, c*, d* are fully complementary to the component strands in the PX molecule. When mixed and incubated at specific temperatures, the complementary strands in the PX and the anti-PX molecules reassociate to form four duplexes a-a*, b-b*, c-c*, and d-d*.

**Figure 2:**
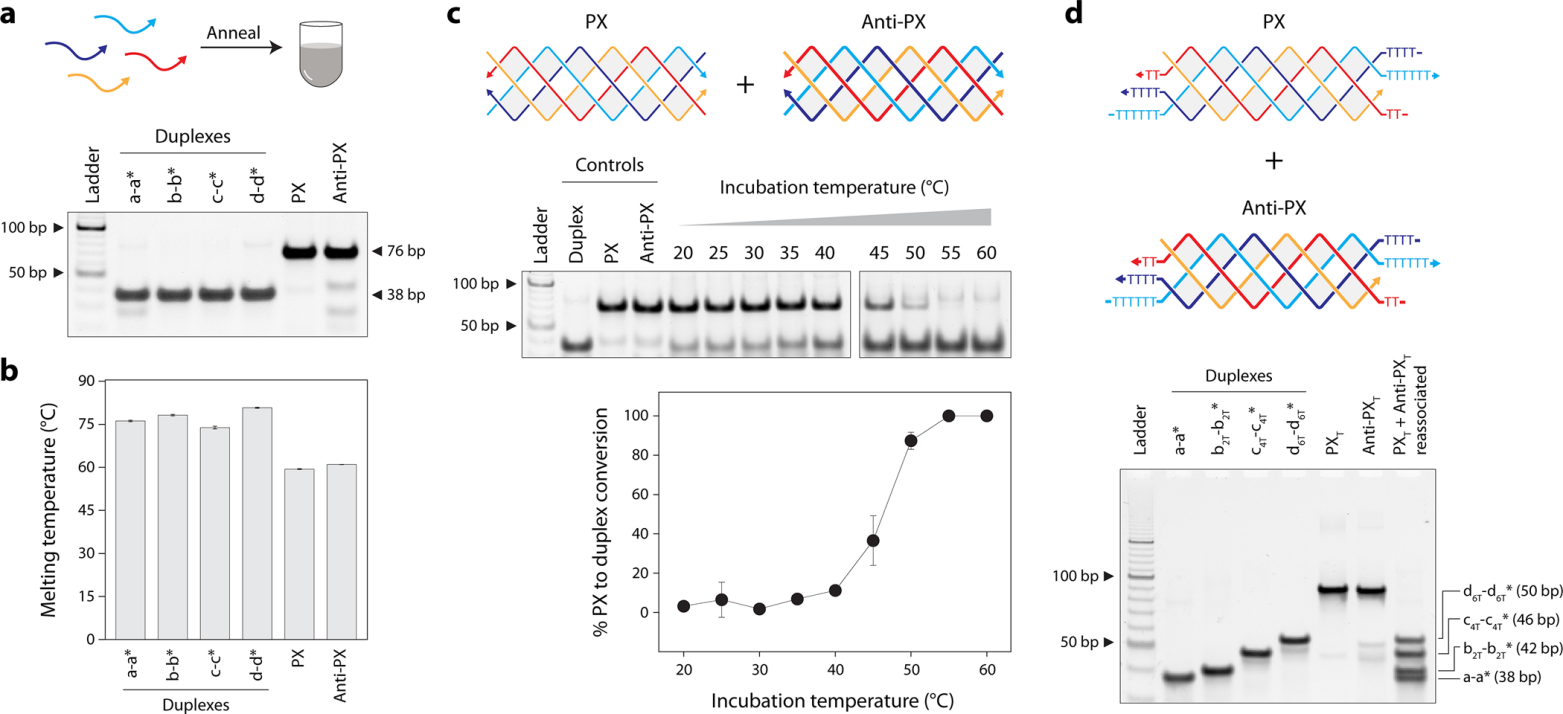
Assembly and reassociation of PX molecules. (a) Nondenaturing gel showing the assembly of the motifs and duplex structures used in this study. (b) UV melting temperatures of the structures. (c) Reassociation of PX and anti-PX molecules into duplexes at different temperatures. Reassociation is quantified as the percent conversion from PX to duplex based on the gel results. (d) PX and anti-PX molecules containing polyT extensions show the formation of four unique duplexes on reassociation.

Our initial notion was to assess whether the PX and anti-PX molecules reassociate at room temperature (20 °C) or physiological temperature (37 °C) to form duplexes if the individual strands in the PX preferred their complements in the anti-PX. However, we observed no reassociation at room temperature. Given the melting temperatures of the PX and anti-PX molecules, we then examined whether the two structures could reassociate at higher temperatures. We assembled the PX and anti-PX, then incubated them together at temperatures ranging from 20 °C to 60 °C for 2 h and analyzed the products on a nondenaturing gel (see Figure 2c and Figure S3). We observed the reassociation of the PX and anti-PX into corresponding duplexes starting at a temperature of ∼40 °C, with the PX-to-duplex conversion increasing with temperature. At a temperature of 55–60 °C, all of the PX and anti-PX were reassociated into duplexes, as also confirmed by dynamic light scattering experiments (Figure S4).

To further demonstrate that the PX and anti-PX molecules reassociate into distinct duplexes when incubated at elevated temperatures, we redesigned the component strands of the PX and anti-PX molecules to include poly-T overhangs at both termini (Figure 2d). To enable clear separation of the resulting duplexes by gel electrophoresis, we extended strands b and b* by 2 thymidines on each end, strands c and c* by 4 thymidines on each end, and strands d and d* by 6 thymidines on each end. The PX and anti-PX molecules with the poly-T ends were incubated together at 60 °C for 3 h to allow complete reassociation. Upon gel electrophoresis, we observed distinct bands corresponding to the expected size of the duplexes a-a* (38 bp), b-b* (42 bp), c-c* (46 bp), and d-d* (50 bp), confirming that incubation of PX and anti-PX at 60 °C leads to reassociation into different, predictable duplex structures.

To ascertain the stability of the PX and anti-PX at these incubation temperatures, we also analyzed the PX and anti-PX incubated individually at these temperatures. While the structures started denaturing at the higher temperatures tested, they were not fully denatured into the component single strands (Figure S5). In combination with the melting experiments, the gel results showed that the PX and anti-PX molecules started to denature at these temperatures, allowing the individual complements to reassociate into duplexes. We note that reassociation of the duplexes into the PX molecules does not occur at the temperatures we tested, possibly due to the higher melting temperature of the duplexes compared to the PX and anti-PX molecules. This is also consistent with prior knowledge that formation of a single four-arm junction^35^ or polycrossover structures^36^ from intact duplexes is unfavorable and that duplexes are preferred over the crossover structures. In fact, when all 8 strands (4 for PX and 4 for anti-PX) are mixed together and annealed, the strands have a strong preference for the duplex structure (Figure S6). We also tested how the mixture of single strands behaves when incubated at different constant temperatures instead of annealing (Figure S7). We noticed partial formation of a PX structure, but the majority of the population was still a duplex, indicating that, at some constant temperatures, the strands partially hybridize with their PX complements. These results indicate that being a part of the PX or anti-PX structure prevents the individual strands from spontaneously reassociating into duplexes. We also tested whether the presence of only one complementary strand would affect the PX structure at different temperatures (Figure S8). Our results indicate that even one strand can displace its complement from the PX structure at higher temperatures.

To improve the reassociation of PX into duplexes at lower temperatures, we added different amounts of formamide to the solution. Formamide has been shown to reduce the DNA melting temperature and is used in isothermal assembly of DNA nanostructures.^37^ We added 10%–40% formamide in the solution containing the PX and anti-PX and incubated the mixture at temperatures ranging from 20 °C to 60 °C (Figure 3a and Figure S9). We observed a trend where the temperature required for the conversion of PX to duplex reduced with increasing formamide concentration in the solution (Figure 3b). Thus, changing the solution conditions allows the complete reassociation of PX molecules into duplexes at temperatures as low as 30 °C.

**Figure 3:**
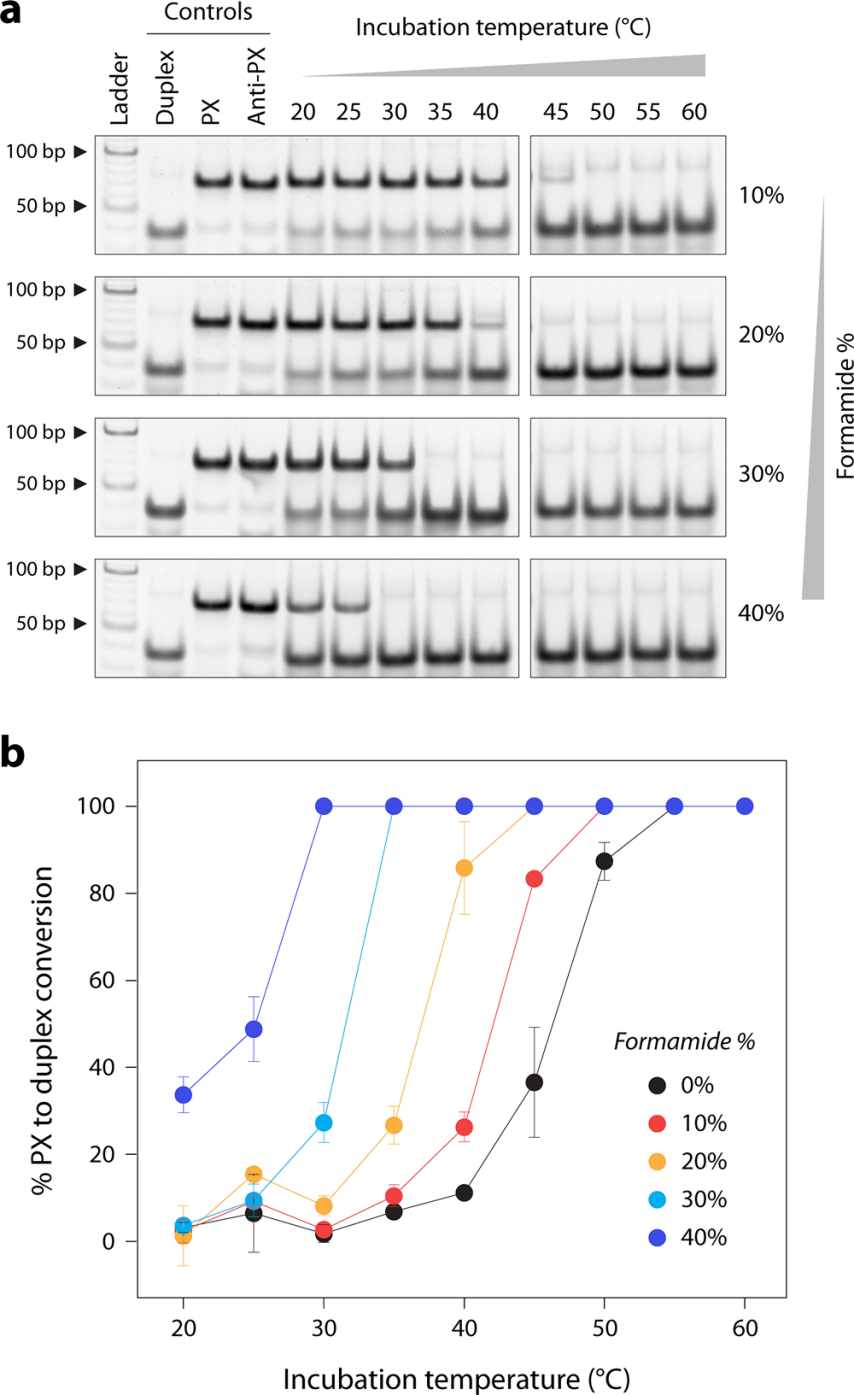
Reducing reassociation temperatures using denaturing agents. (a) Nondenaturing gels showing the reassociation of PX and anti-PX molecules into duplexes in the presence of different amounts of formamide. (b) Quantified results show a steady decrease in the temperature required for reassociation with increasing formamide concentrations.

Next, we used the reassociation of PX to duplex to demonstrate tunable biostability of DNA structures. Our earlier work showed that PX DNA has exceptional nuclease resistance when tested against nucleases and body fluids.^33^ Here, we incubated the PX/anti-PX mixture with different amounts of DNase I and observed minimal degradation over 30 min (Figure 4 and Figure S10). We performed the DNase I assay at 20 °C so that there is no reassociation of the PX and anti-PX molecules, a temperature at which DNase I still shows activity. We then used the same starting batch of PX/anti-PX mixture and incubated it at 60 °C to ensure complete reassociation into duplexes. After the solution reached 20 °C, we incubated the reassociated duplexes with the same amounts of DNase I as before and observed that the duplexes degraded rapidly (Figure 4 and Figure S10). We show that structures with high biostability (PX and anti-PX) can be reassociated into structures with weak biostability (duplexes), allowing one to introduce sequence- and temperature-based tunability between structures of vastly different biostability.

**Figure 4:**
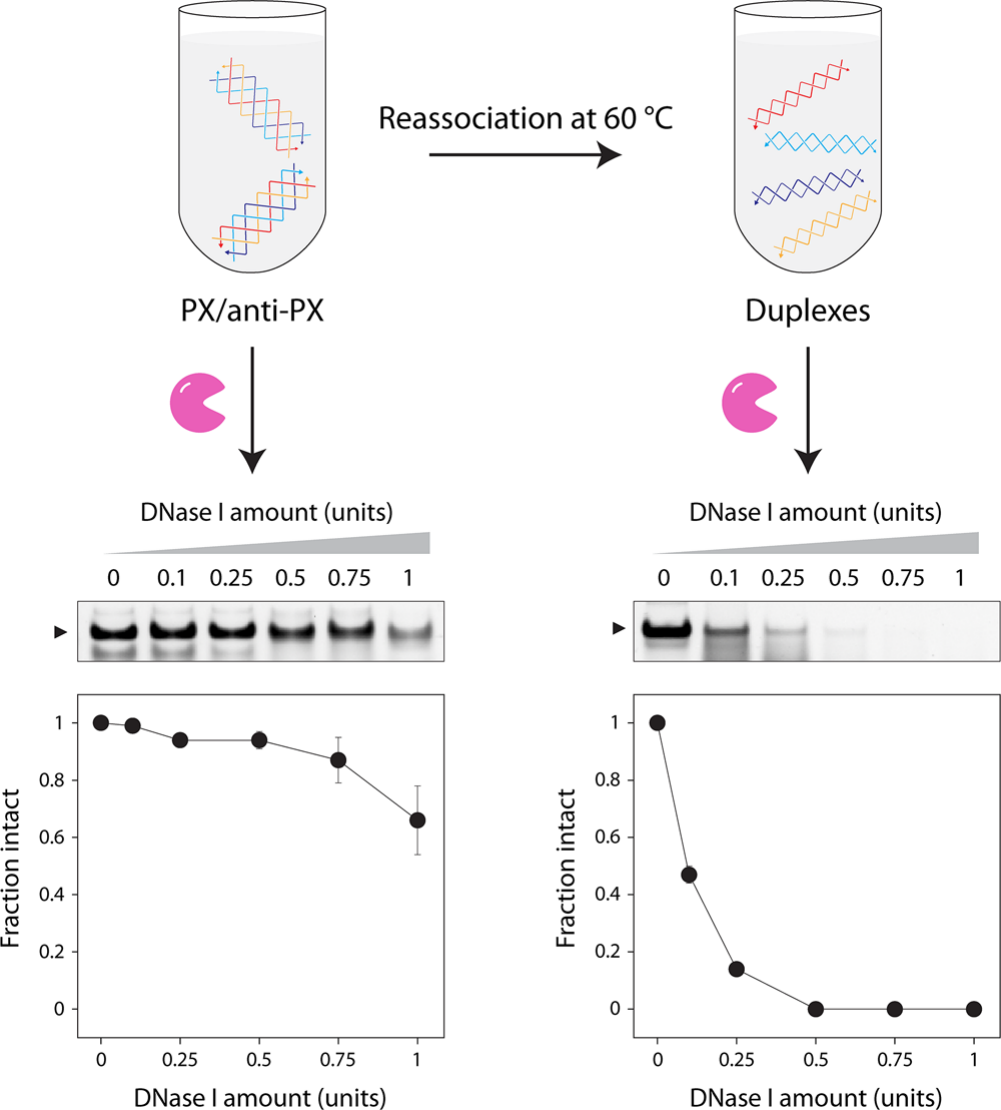
Tunable biostability. (Top) Illustration showing the DNase I treatment of PX/anti-PX mixture, and the reassociation of PX/anti-PX into duplex for DNase I treatment. (Bottom) Nondenaturing gel and quantified results of DNase I treated samples. PX/anti-PX molecules are nuclease resistant, showing only partial degradation over the range of nuclease concentrations tested, whereas the duplexes degrade completely.

Finally, we tested the effect of the number of crossovers in the structure on their reassociation temperature. We chose the double crossover (DX) motif that contains two crossovers and the juxtaposed crossover (JX) structure, a topoisomer of PX, that contains four crossovers (Figure 5a). Similar to the PX and anti-PX, we designed anti-DX and anti-JX structures, where the component strands in anti-DX and anti-JX are fully complementary to the component strands in DX and JX, respectively (Figure S11 and S12). We incubated the DX/anti-DX and JX/anti-JX mixtures at different temperatures and observed that the two structures required different temperature ranges to reassociate into duplexes compared to the PX (see Figures 5b and 5c and Figure S13). We found that the trend in the reassociation temperatures for these structures indicates a dependency on their thermal stability, rather than the number of crossovers in the structures. We observed that DX > PX > JX, when comparing 50% reassociation into duplexes; this is a trend that is consistent with the melting temperatures of these structures (Figure S14).

**Figure 5:**
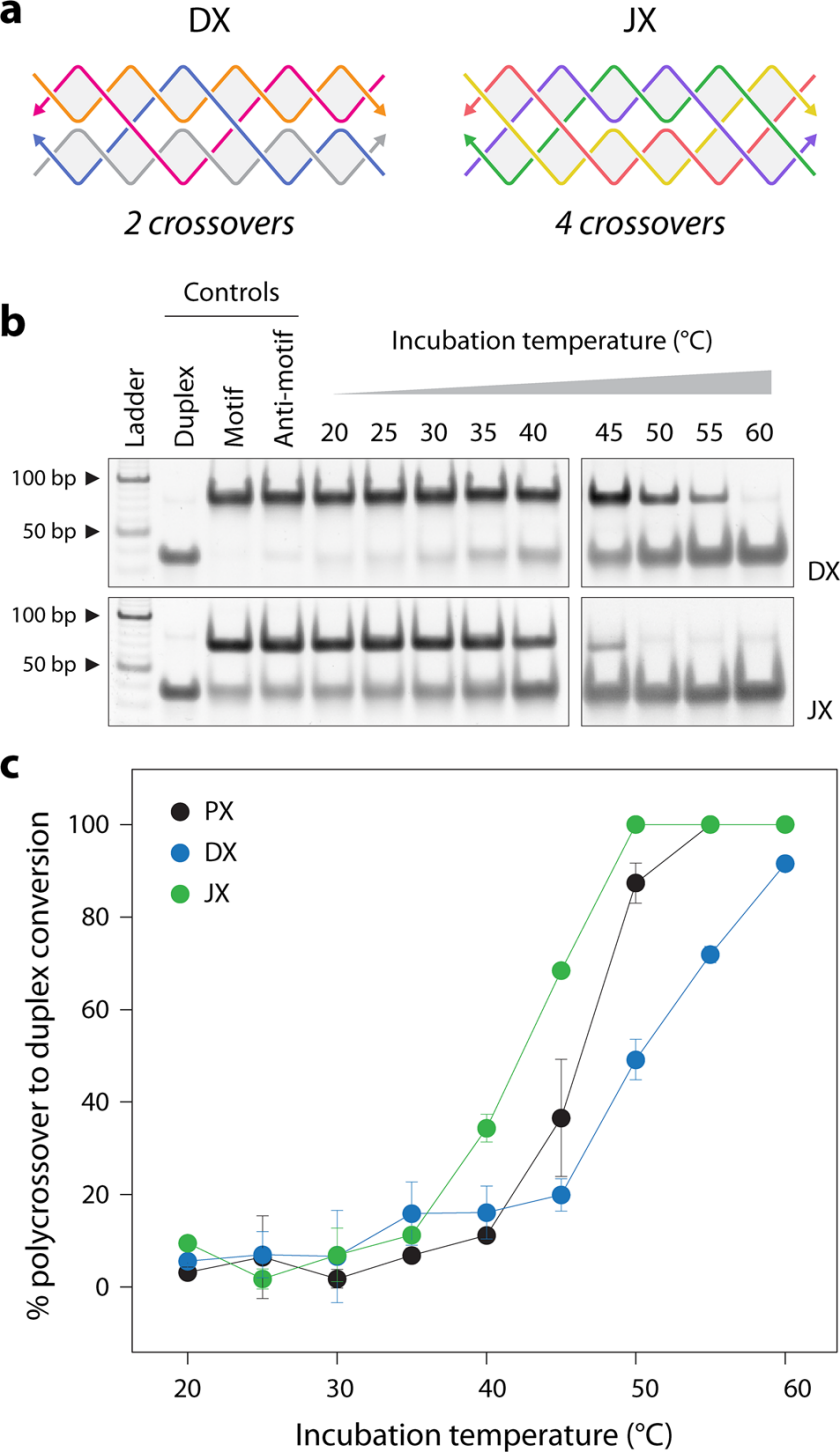
Reassociation of polycrossover DNA molecules into duplexes. (a) Scheme of a double crossover (DX) motif and a juxtaposed crossover (JX) motif that contains four crossovers. (b) Nondenaturing gels and (c) quantified results showing the reassociation of the polycrossover molecules into duplexes at different temperatures.

In this work, we demonstrate that multistranded, tightly knit DNA motifs of a specific sequence composition can reassociate into duplexes. We show that reassociation can be achieved in a temperature-dependent manner, allowing the conversion of structures with vastly different nuclease resistance levels. The reassociation is triggered in part by the partial melting of the PX complexes, which allows the component strands within a structure to be displaced by its complement in the antistructure. While high temperature dependence is usually not preferred, operation of DNA nanostructures using heat as a stimuli is a useful feature, as shown in the cyclic transition of DNA origami dimers with fluctuating temperatures.^38^ For applications that require lower operating temperatures, we show that addition of formamide allows the reassociation temperature to go down by as much as ∼25 °C. Formamide has been used to fold the two individual strands of a double-stranded DNA scaffold into independent structures,^39^ in the isothermal assembly of DNA polyhedra^40^ and origami,^41,42^ and to accelerate the kinetics of DNA array transformation.^43^ Our strategy could also be combined with functional DNA modules that can assemble and reassociate in the presence of specific ligands.^44^ Since temperature is a key requirement, activation and reassociation can be achieved by local heating as in nanoscale DNA heat engines that perform physical tasks such as moving beads on an origami.^45^

Structures such as the DX and PX DNA motifs are shown to be involved in biological processes such as homologous recombination.^32,46^ Thus, our reassociation strategy has the potential to be adapted for biological applications where physiological temperatures can reassociate structures for different functionalities. Our prior work has also shown that PX and DX motifs do not affect cell viability or cause adverse immune response, making these structures useful in biological applications.^33,47^ Similar to split aptamers and split enzymes that become active upon reassociation,^48^ nanostructures that combine to form “active” duplexes might be useful in biosensing and drug delivery applications. In the context of dynamic DNA nanotechnology, our work provides a route to create a group of nanostructures that can compute biomolecular information, resulting in reassociation into a different set of nanostructures, with properties that are useful in several biomedical applications.

## Supplementary Material



## Notes

Experimental procedures, additional results and DNA sequences used (PDF)

## References

[1] Chidchob P., Sleiman H. F. (2018). Recent Advances in DNA Nanotechnology. Curr. Opin. Chem. Biol..

[2] Juul S., Iacovelli F., Falconi M., Kragh S. L., Christensen B., Frøhlich R., Franch O., Kristoffersen E. L., Stougaard M., Leong K. W., Ho Y.-P., Sørensen E. S., Birkedal V., Desideri A., Knudsen B. R. (2013). Temperature-Controlled
Encapsulation and Release of an Active Enzyme in the Cavity of a Self-Assembled
DNA Nanocage. ACS Nano.

[3] Kohman R. E., Han X. (2015). Light Sensitization
of DNA Nanostructures via Incorporation of Photo-Cleavable
Spacers. Chem. Commun..

[4] Ji W., Li D., Lai W., Yao X., Alam Md. F., Zhang W., Pei H., Li L., Chandrasekaran A. R. (2019). pH-Operated Triplex DNA Device on
MoS2 Nanosheets. Langmuir.

[5] Chandrasekaran A. R., Halvorsen K. (2019). Controlled
Disassembly of a DNA Tetrahedron Using Strand
Displacement. Nanoscale Adv..

[6] Li S., Jiang Q., Liu S., Zhang Y., Tian Y., Song C., Wang J., Zou Y., Anderson G. J., Han J.-Y., Chang Y., Liu Y., Zhang C., Chen L., Zhou G., Nie G., Yan H., Ding B., Zhao Y. (2018). A DNA Nanorobot Functions as a Cancer
Therapeutic in Response to a Molecular Trigger in Vivo. Nat. Biotechnol..

[7] Ranallo S., Sorrentino D., Ricci F. (2019). Orthogonal Regulation of DNA Nanostructure
Self-Assembly and Disassembly Using Antibodies. Nat. Commun..

[8] Simmel F. C. (2023). Nucleic
Acid Strand Displacement – from DNA Nanotechnology to Translational
Regulation. RNA Biol..

[9] Talbot H., Chandrasekaran A. R. (2025). Mismatch-Induced
Toehold-Free Strand Displacement Used
to Control a DNA Nanodevice. ACS Synth. Biol..

[10] Chandrasekaran A. R. (2024). A DNA Rotary
Nanodevice Operated by Enzyme-Initiated Strand Resetting. Chem. Commun..

[11] Montagud-Martínez R., Heras-Hernández M., Goiriz L., Daròs J.-A., Rodrigo G. (2021). CRISPR-Mediated Strand
Displacement Logic Circuits
with Toehold-Free DNA. ACS Synth. Biol..

[12] Kang H., Lin T., Xu X., Jia Q.-S., Lakerveld R., Wei B. (2021). DNA Dynamics and Computation Based on Toehold-Free Strand Displacement. Nat. Commun..

[13] Madhanagopal B. R., Talbot H., Rodriguez A., Louis J. M., Zeghal H., Vangaveti S., Reddy K., Chandrasekaran A. R. (2024). The Unusual
Structural Properties and Potential Biological Relevance of Switchback
DNA. Nat. Commun..

[14] Zuo H., Mao C. (2019). A Minimalist’s
Approach for DNA Nanoconstructions. Adv. Drug
Delivery Rev..

[15] Liu H., Chen Y., He Y., Ribbe A. E., Mao C. (2006). Approaching
The Limit: Can One DNA Oligonucleotide Assemble into Large Nanostructures?. Angew. Chem., Int. Ed..

[16] Tian C., Zhang C., Li X., Hao C., Ye S., Mao C. (2014). Approaching the Limit: Can One DNA
Strand Assemble into Defined Nanostructures?. Langmuir.

[17] Zhao J., Zhang C., Lu B., Sha R., Noinaj N., Mao C. (2023). Divergence and Convergence: Complexity Emerges in Crystal Engineering
from an 8-Mer DNA. J. Am. Chem. Soc..

[18] Nie Z., Li X., Li Y., Tian C., Wang P., Mao C. (2013). Self-Assembly
of DNA Nanoprisms with Only Two Component Strands. Chem. Commun..

[19] Zheng M., Li Z., Liu L., Li M., Paluzzi V. E., Hyun
Choi J., Mao C. (2021). Kinetic DNA Self-Assembly: Simultaneously Co-Folding
Complementary DNA Strands into Identical Nanostructures. J. Am. Chem. Soc..

[20] Beasock D., Ha A., Halman J., Panigaj M., Wang J., Dokholyan N. V., Afonin K. A. (2023). Break to Build: Isothermal Assembly of Nucleic Acid
Nanoparticles (NANPs) via Enzymatic Degradation. Bioconjugate Chem..

[21] Halman J. R., Satterwhite E., Roark B., Chandler M., Viard M., Ivanina A., Bindewald E., Kasprzak W. K., Panigaj M., Bui M. N. (2017). Functionally-Interdependent Shape-Switching Nanoparticles
with Controllable Properties. Nucleic Acids
Res..

[22] Ke W., Hong E., Saito R. F., Rangel M. C., Wang J., Viard M., Richardson M., Khisamutdinov E. F., Panigaj M., Dokholyan N. V., Chammas R., Dobrovolskaia M. A., Afonin K. A. (2019). RNA–DNA Fibers and Polygons with Controlled
Immunorecognition Activate RNAi, FRET and Transcriptional Regulation
of NF-κB in Human Cells. Nucleic Acids
Res..

[23] Afonin K. A., Viard M., Martins A. N., Lockett S. J., Maciag A. E., Freed E. O., Heldman E., Jaeger L., Blumenthal R., Shapiro B. A. (2013). Activation of Different Split Functionalities on Re-Association
of RNA–DNA Hybrids. Nat. Nanotechnol..

[24] Hartung J., McCann N., Doe E., Hayth H., Benkato K., Johnson M. B., Viard M., Afonin K. A., Khisamutdinov E. F. (2023). Toehold-Mediated
Shape Transition of Nucleic Acid Nanoparticles. ACS Appl. Mater. Interfaces.

[25] Chandler M., Afonin K. A. (2019). Smart-Responsive
Nucleic Acid Nanoparticles (NANPs)
with the Potential to Modulate Immune Behavior. Nanomaterials.

[26] Wang X., Chandrasekaran A. R., Shen Z., Ohayon Y. P., Wang T., Kizer M. E., Sha R., Mao C., Yan H., Zhang X., Liao S., Ding B., Chakraborty B., Jonoska N., Niu D., Gu H., Chao J., Gao X., Li Y., Ciengshin T., Seeman N. C. (2019). Paranemic Crossover
DNA: There and Back Again. Chem. Rev..

[27] Ohayon Y. P., Sha R., Flint O., Liu W., Chakraborty B., Subramanian H. K. K., Zheng J., Chandrasekaran A. R., Abdallah H. O., Wang X., Zhang X., Seeman N. C. (2015). Covalent
Linkage of One-Dimensional DNA Arrays Bonded by Paranemic Cohesion. ACS Nano.

[28] Shen W., Liu Q., Ding B., Shen Z., Zhu C., Mao C. (2016). The Study
of the Paranemic Crossover (PX) Motif in the Context of Self-Assembly
of DNA 2D Crystals. Org. Biomol. Chem..

[29] Zhang X., Yan H., Shen Z., Seeman N. C. (2002). Paranemic Cohesion of Topologically-Closed
DNA Molecules. J. Am. Chem. Soc..

[30] Qi X., Zhang F., Su Z., Jiang S., Han D., Ding B., Liu Y., Chiu W., Yin P., Yan H. (2018). Programming Molecular
Topologies from Single-Stranded Nucleic Acids. Nat. Commun..

[31] Han D., Qi X., Myhrvold C., Wang B., Dai M., Jiang S., Bates M., Liu Y., An B., Zhang F., Yan H., Yin P. (2017). Single-Stranded DNA and RNA Origami. Science.

[32] Wang X., Zhang X., Mao C., Seeman N. C. (2010). Double-Stranded
DNA Homology Produces a Physical Signature. Proc. Natl. Acad. Sci. U.S.A..

[33] Chandrasekaran A. R., Vilcapoma J., Dey P., Wong-Deyrup S. W., Dey B. K., Halvorsen K. (2020). Exceptional
Nuclease Resistance of
Paranemic Crossover (PX) DNA and Crossover-Dependent Biostability
of DNA Motifs. J. Am. Chem. Soc..

[34] Shen Z., Yan H., Wang T., Seeman N. C. (2004). Paranemic Crossover DNA: A Generalized
Holliday Structure with Applications in Nanotechnology. J. Am. Chem. Soc..

[35] Marky L. A., Kallenbach N. R., McDonough K. A., Seeman N. C., Breslauer K. J. (1987). The Melting
Behavior of a DNA Junction Structure: A Calorimetric and Spectroscopic
Study. Biopolymers.

[36] Spink C. H., Ding L., Yang Q., Sheardy R. D., Seeman N. C. (2009). Thermodynamics
of Forming a Parallel DNA Crossover. Biophys.
J..

[37] Chandrasekaran A. R. (2025). Isothermal
Assembly of DNA Nanostructures. Chem. Commun..

[38] Chen Z., Chen K., Xie C., Liao K., Xu F., Pan L. (2023). Cyclic Transitions
of DNA Origami Dimers Driven by Thermal Cycling. Nanotechnology.

[39] Högberg B., Liedl T., Shih W. M. (2009). Folding DNA Origami
from a Double-Stranded
Source of Scaffold. J. Am. Chem. Soc..

[40] Wang B., Song L., Jin B., Deng N., Wu X., He J., Deng Z., Li Y. (2019). Base-Sequence-Independent Efficient
Redox Switching of Self-Assembled DNA Nanocages. ChemBioChem..

[41] Zhang Z., Song J., Besenbacher F., Dong M., Gothelf K. V. (2013). Self-Assembly
of DNA Origami and Single-Stranded Tile Structures at Room Temperature. Angew. Chem., Int. Ed..

[42] Jungmann R., Liedl T., Sobey T. L., Shih W., Simmel F. C. (2008). Isothermal
Assembly of DNA Origami Structures Using Denaturing Agents. J. Am. Chem. Soc..

[43] Song J., Li Z., Wang P., Meyer T., Mao C., Ke Y. (2017). Reconfiguration
of DNA Molecular Arrays Driven by Information Relay. Science.

[44] Chen S., Hermann T. (2021). RNA–DNA Hybrid
Nanoshape Synthesis by Facile
Module Exchange. J. Am. Chem. Soc..

[45] Wang K., Hardikar A., Chen W., Huang Q., Guo B., Zhu G., Ni H., Sha R., Seeman N. C., Chaikin P. M. (2025). Control
and Synchronization of Rapid Nanoscale DNA Heat Engine by Local Heating. Sci. Adv..

[46] Wright W. D., Shah S. S., Heyer W.-D. (2018). Homologous
Recombination and the
Repair of DNA Double-Strand Breaks. J. Biol.
Chem..

[47] Rodriguez A., Madhanagopal B. R., Sarkar K., Nowzari Z., Mathivanan J., Talbot H., Patel A., Morya V., Halvorsen K., Vangaveti S., Berglund J. A., Chandrasekaran A. R. (2025). Counterions
Influence the Isothermal Self-Assembly of DNA Nanostructures. Sci. Adv..

[48] Debiais M., Lelievre A., Smietana M., Müller S. (2020). Splitting
Aptamers and Nucleic Acid Enzymes for the Development of Advanced
Biosensors. Nucleic Acids Res..

