# Supplementary material for: Controlled Reassociation of Multistranded, Polycrossover DNA Molecules into Double Helices
Source: Nano Lett. 2025 Nov 3;25(47):16658–63. doi: 10.1021/acs.nanolett.5c04286 (PMC12614193; doi:10.1021/acs.nanolett.5c04286)
Supplement: Supplementary file 1 [file nl5c04286_si_001.pdf]

Supporting information

Controlled reassociation of multi-stranded, polycrossover DNA molecules into double helices

Nada Kabbara,[1] Lauren A. Anderson,[1] Shubhajit Singha,[2] and Arun Richard Chandrasekaran[1,3,*]

[1]Department of Nanoscale Science and Engineering, University at Albany, State University of New York, Albany, NY, USA.

[2]Department of Chemistry, University at Albany, State University of New York, Albany, NY, USA.

[3]The RNA Institute, University at Albany, State University of New York, Albany, NY, USA.

*Correspondence: arun@albany.edu

MATERIALS AND METHODS

Preparation of DNA complexes

DNA strands were purchased from Integrated DNA Technologies (IDT). Full sequences are listed in Table S1. PX, DX and JX complexes were prepared by mixing the component DNA strands in equal ratios in Tris-Acetic-EDTA buffer containing 40 mM Tris base (pH 8), 20 mM acetic acid, 2 mM EDTA, and 12.5 mM magnesium acetate (1× TAE-Mg^{2+}). Samples were placed in a beaker containing 2 liters of deionized water heated to 90 °C, then placed in a Styrofoam box to cool to 20 °C over the course of 2 days. For duplexes, strands were combined in equal ratios in 1× TAE-Mg^{2+} buffer and annealed using a thermocycler from 90 °C to 20 °C over 30 minutes.

Structure reassociation

Assembled PX and anti-PX structures were mixed in equal molar ratios and incubated at different temperatures in a BioRad thermal cycler. The same procedure was followed for the DX and JX structures. Percent conversion from PX (or DX or JX) to duplex was calculated as the reduction in intensity of the band corresponding to multi-stranded structures (PX, DX or JX), and normalized to the anti-structure control band intensity. For reassociation in formamide, assembled PX and anti-PX structures were mixed in equal molar ratios in solution containing formamide (concentrations discussed in main text) and incubated at different temperatures in a BioRad thermal cycler.

Nuclease degradation assay

For PX samples, the PX and anti-PX were mixed at a final concentration of 0.5 µM and used for the DNase I assay (DNase I, New England Biolabs). To convert the PX/anti-PX mixture into duplexes, the PX and anti-PX mixture was heated at 60 °C for 3 h and the resulting sample was used in the DNase I assay. DNA samples were first mixed with DNase I reaction buffer (provided by the vendor) to a final 1× concentration. Enzyme dilutions were made in nuclease-free water. For the nuclease degradation assay, 1 µl of the enzyme was added to 10 µl of the DNA sample containing the reaction buffer and incubated at 20 °C for 30 min. Incubated samples were mixed with gel loading dye containing bromophenol blue and 1× TAE-Mg^{2+} buffer and run on non-denaturing gels to analyze degradation. Degradation profiles were obtained by normalizing the band intensity corresponding to the structure (at each enzyme concentration) to the control DNA lane without any enzyme.

Gel electrophoresis

Non-denaturing gels were prepared using 19:1 acrylamide/bisacrylamide (National Diagnostics). Samples were mixed with loading dye containing bromophenol blue and glycerol prior to loading. Gels were run at a constant voltage at 4 °C in 1× TAE-Mg^{2+} running buffer. Gels were stained in 0.5× GelRed (Biotium), imaged using a Bio-Rad Gel Doc XR+ and analyzed using ImageLab software.

UV melting

UV melting experiments were performed on a Cary 3500 UV-Visible Spectrophotometer (Agilent) using 1 μM DNA concentration. Absorbance at 260 nm was recorded while samples were heated from 15 °C to 95 °C at a rate of 0.5 °C/min. This data was normalized to 0-1 and fitted to the Boltzmann curve using OriginPro. Melting temperatures were determined from the first derivative of the fitted melting curves.

Dynamic light scattering (DLS)

The hydrodynamic diameter of PX DNA and reassociated duplexes were determined by DLS using a Zetasizer Nano-ZS (Malvern, USA) instrument equipped with a non-invasive backscatter (NIBS) detector. Measurements were performed at 25 °C with an equilibration time of 120 seconds prior to data acquisition. PX and anti-PX samples were prepared at 250 nM DNA concentration and a total sample volume of 1 ml was used for each run. For reassociation, 250 nM PX and anti-PX molecules were mixed and incubated at 60 °C for 3 h. The reassociated sample was used as is for DLS of the resulting duplexes.

Strand name	Sequence
PX1	GTGGTATCATCAATGCTATGTGTAGGCTTAGACCTGAG
PX2	ACTAGGTCGCAACAGACACAATACTTGACCGAATCACT
PX3	AGTGAGTCTAACAAGTCACATATCTGTGATGATCTAGT
PX4	CTCAGTTCGGTGCCTAATTGTGGCATTTGCGACACCAC
Anti-PX1	CTCAGGTCTAAGCCTACACATAGCATTGATGATACCAC
Anti-PX2	AGTGATTCGGTCAAGTATTGTGTCTGTTGCGACCTAGT
Anti-PX3	ACTAGATCATCACAGATATGTGACTTGTTAGACTCACT
Anti-PX4	GTGGTGTCGCAAATGCCACAATTAGGCACCGAACTGAG
JX1	GTGGTATCATCAATGCCACAATACTTGACCGAATCACT
JX2	ACTAGGTCGCAACAGATATGTGTAGGCTTAGACCTGAG
JX3	AGTGAGTCTAACAAGTATTGTGGCATTTGCGACACCAC
JX4	CTCAGTTCGGTGCCTACACATATCTGTGATGATCTAGT
Anti-JX1	AGTGATTCGGTCAAGTATTGTGGCATTGATGATACCAC
Anti-JX2	CTCAGGTCTAAGCCTACACATATCTGTTGCGACCTAGT
Anti-JX3	GTGGTGTCGCAAATGCCACAATACTTGTTAGACTCACT
Anti-JX4	ACTAGATCATCACAGATATGTGTAGGCACCGAACTGAG
DX1	AGTGATTCGGTGCCTACACATATCTGTTGCGACACCAC
DX2	GTGGTGTCGCAACAGACACAATACTTCACCGAATCACT
DX3	ACTAGATCATCAATGCTATGTGTAGGGTTAGACCTGAG
DX4	CTCAGGTCTAACAAGTATTGTGGCATTGATGATCTAGT
Anti-DX1	GTGGTGTCGCAACAGATATGTGTAGGCACCGAATCACT
Anti-DX2	AGTGATTCGGTGAAGTATTGTGTCTGTTGCGACACCAC
Anti-DX3	CTCAGGTCTAACCCTACACATAGCATTGATGATCTAGT
Anti-DX4	ACTAGATCATCAATGCCACAATACTTGTTAGACCTGAG
PX2$_{2T}$	TTACTAGGTCGCAACAGACACAATACTTGACCGAATCACTTT
PX3$_{4T}$	TTTTAGTGAGTCTAACAAGTCACATATCTGTGATGATCTAGTTTTT
PX4$_{6T}$	TTTTTTCTCAGTTCGGTGCCTAATTGTGGCATTTGCGACACCACTTTTTT
Anti-PX2$_{2T}$	TTAGTGATTCGGTCAAGTATTGTGTCTGTTGCGACCTAGTTT
Anti-PX3$_{4T}$	TTTTACTAGATCATCACAGATATGTGACTTGTTAGACTCACTTTTT
Anti-PX4$_{6T}$	TTTTTTGTGGTGTCGCAAATGCCACAATTAGGCACCGAACTGAGTTTTTT

Table S1. Sequences used in the study (written 5' to 3').

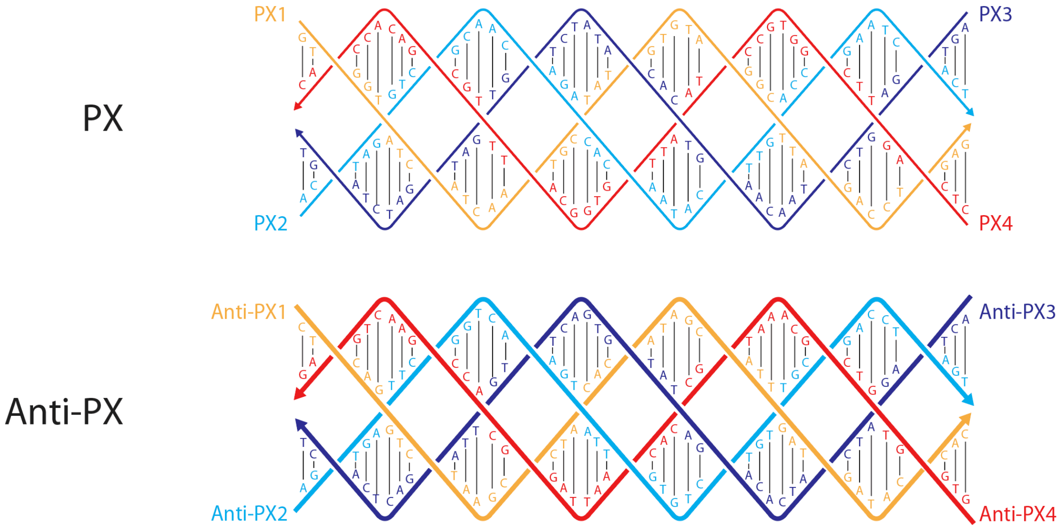

Figure S1. Sequence diagram for PX and anti-PX.

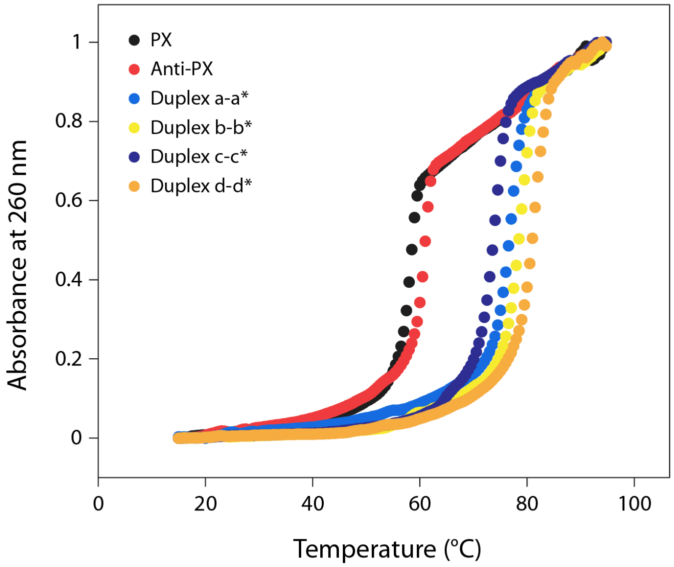

Figure S2. UV melting curves of PX and duplex molecules.

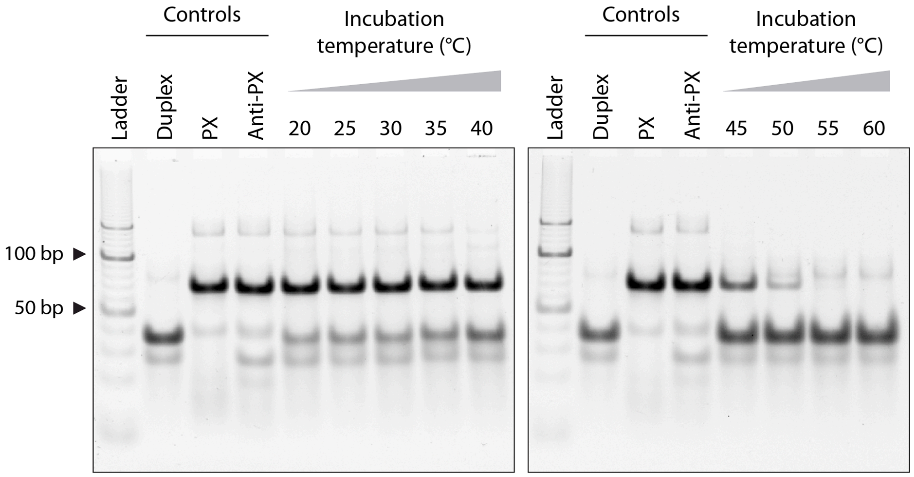

Figure S3. Non-denaturing gels showing the reassociation of PX and anti-PX in 1× TAE-Mg^{2+} buffer at different temperatures. Full images of gels shown in Figure 2c.

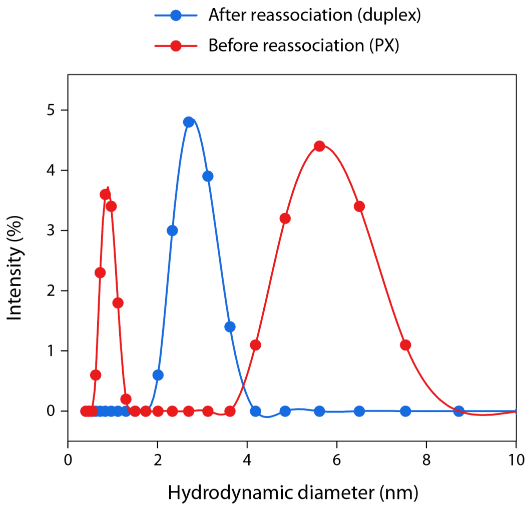

Figure S4. Size-distribution profile obtained by dynamic light scattering (DLS) showing the conversion of PX structures into duplexes after reassociation at 60 °C for 3 h.

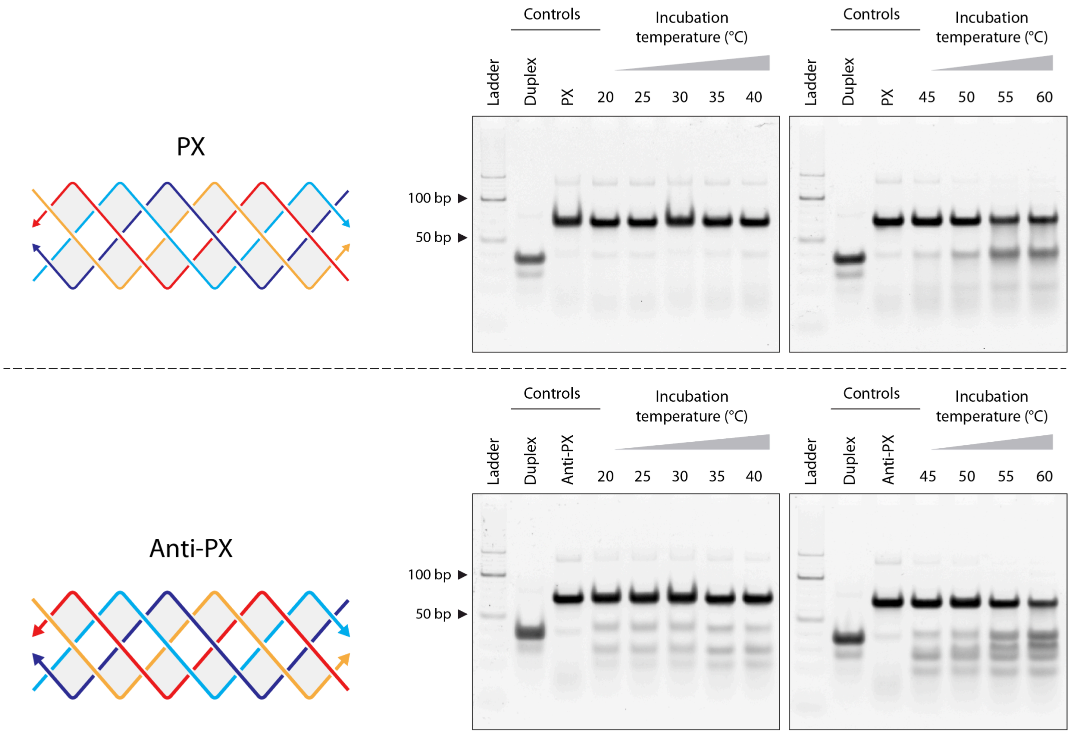

Figure S5. Non-denaturing gels showing stability of PX and anti-PX at different temperatures.

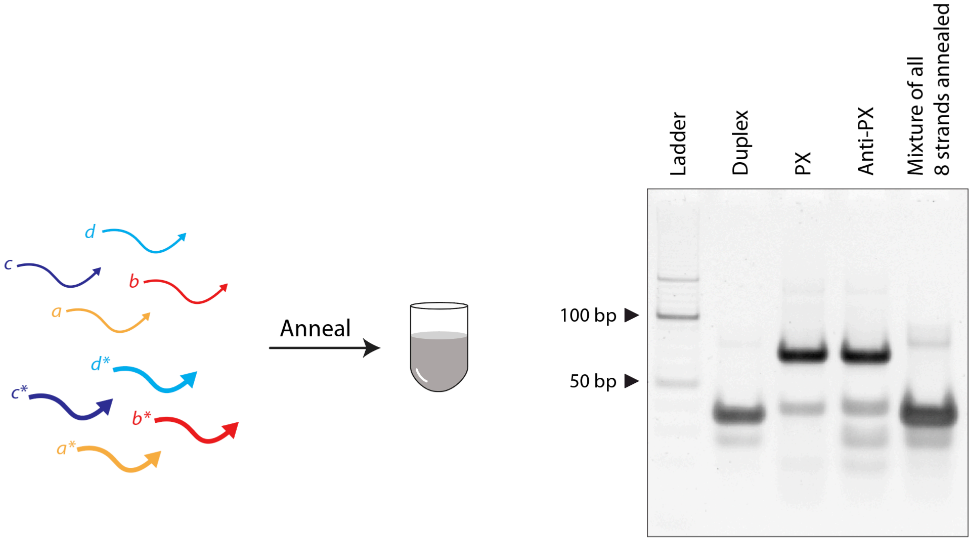

Figure S6. Non-denaturing gels showing preference towards duplex structure when all 8 strands are present in the mixture as single strands and annealed.

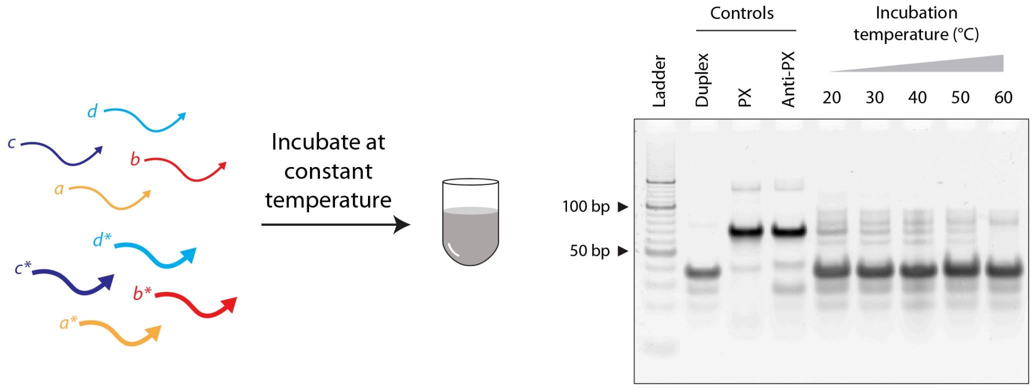

Figure S7. Component strands of the PX and anti-PX were mixed together and incubated at different temperatures for 2 h.

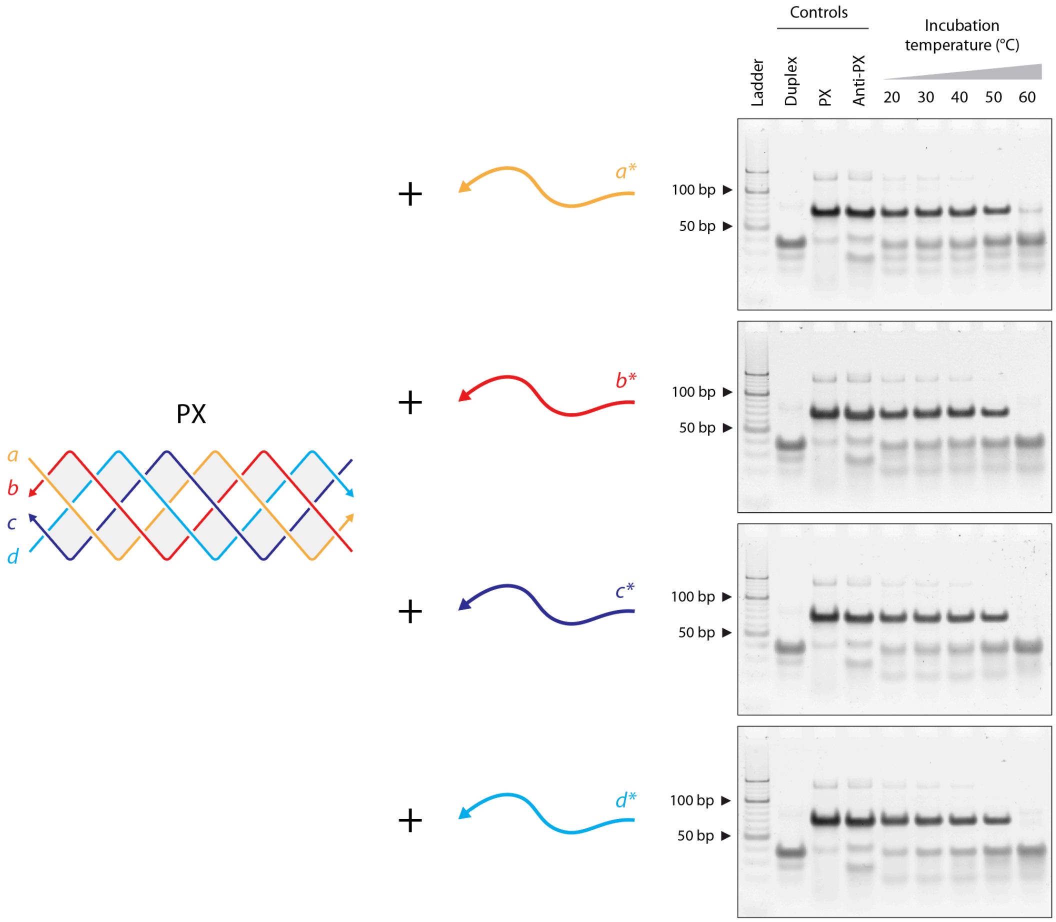

Figure S8. Effect of the addition of only one complementary strand to a pre-annealed PX.

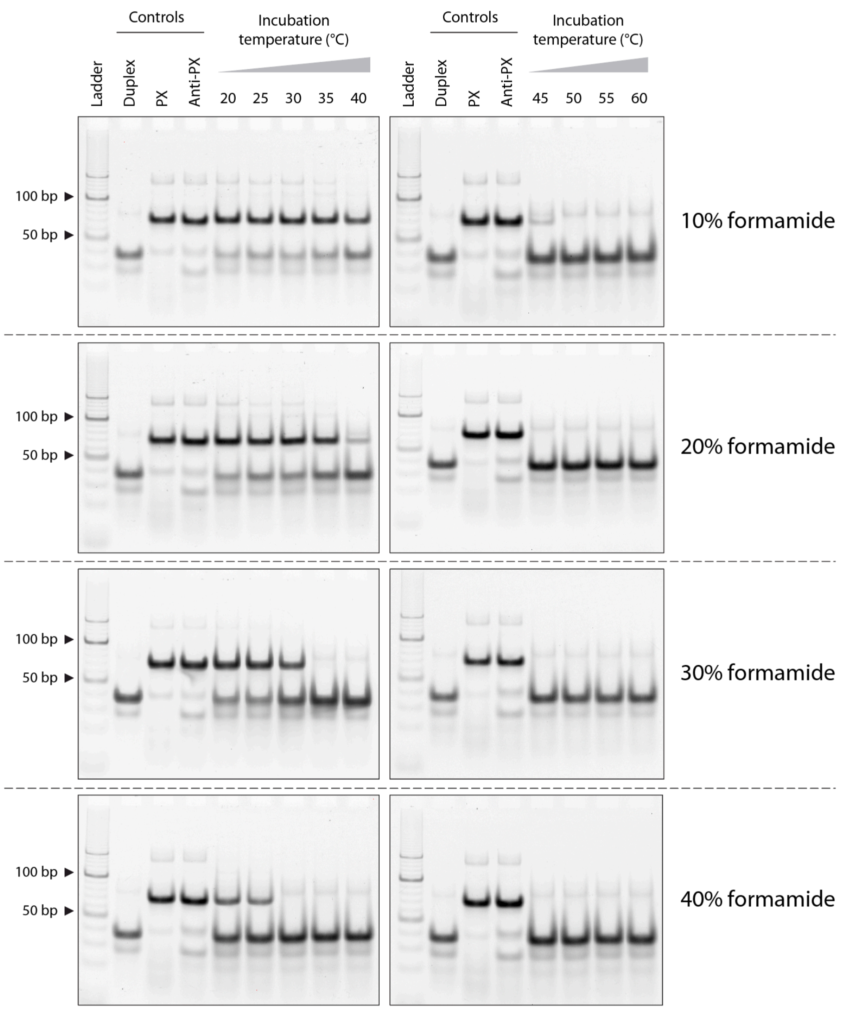

Figure S9. Non-denaturing gels showing reassociation of PX and anti-PX at different temperatures in 1× TAE-Mg^{2+} buffer containing 10-40% formamide. Full images of gels shown in Figure 3a.

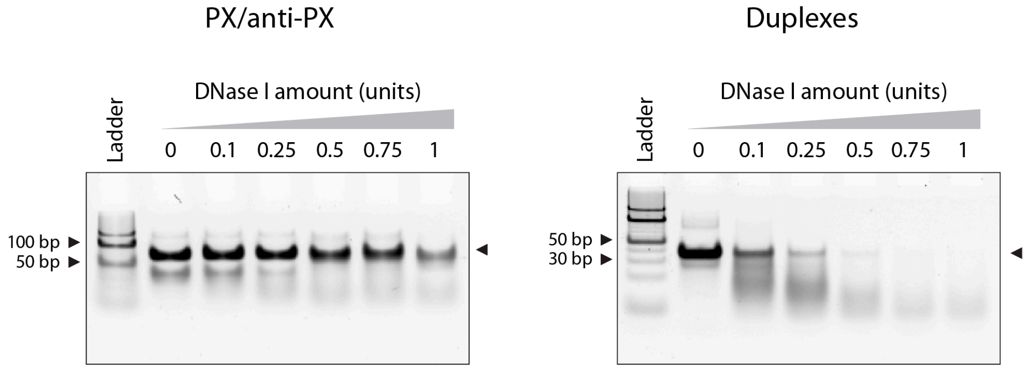

Figure S10. Non-denaturing gels showing DNase I degradation of PX/anti-PX samples (left) and reassociated duplexes (right). Full images of gels shown in Figure 4.

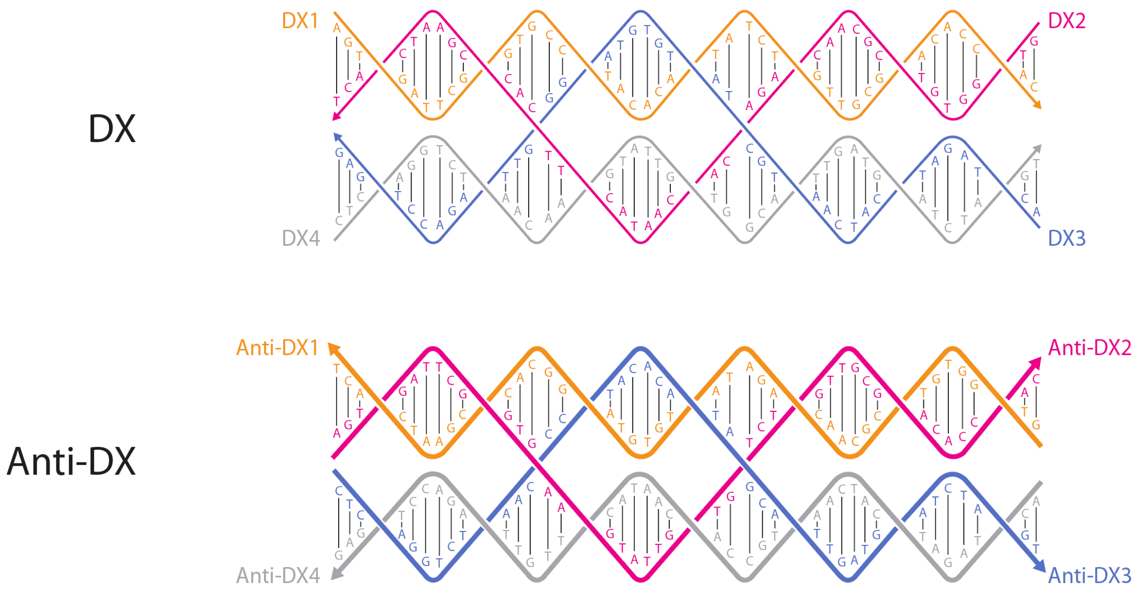

Figure S11. Sequence diagram for DX and anti-DX structures.

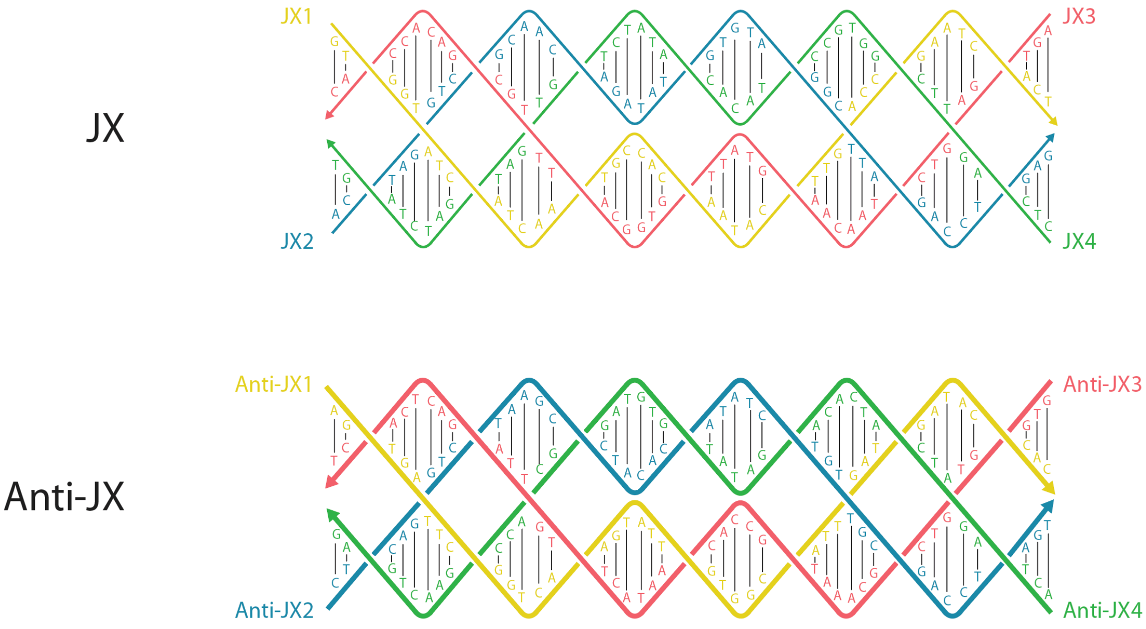

Figure S12. Sequence diagram for JX and anti-JX structures.

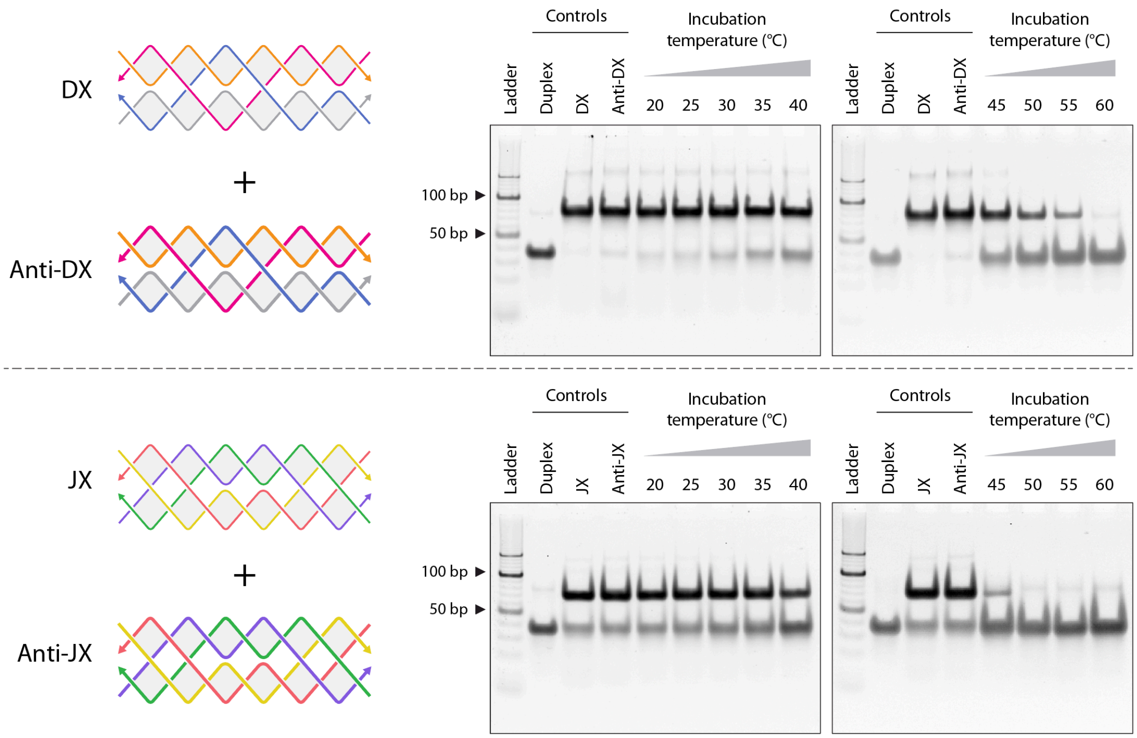

Figure S13. Non-denaturing gels showing the reassociation of DX and anti-DX (top) and JX and anti-JX (bottom) in 1× TAE-Mg^{2+} buffer at different temperatures. Full images of gels shown in Figure 5b.

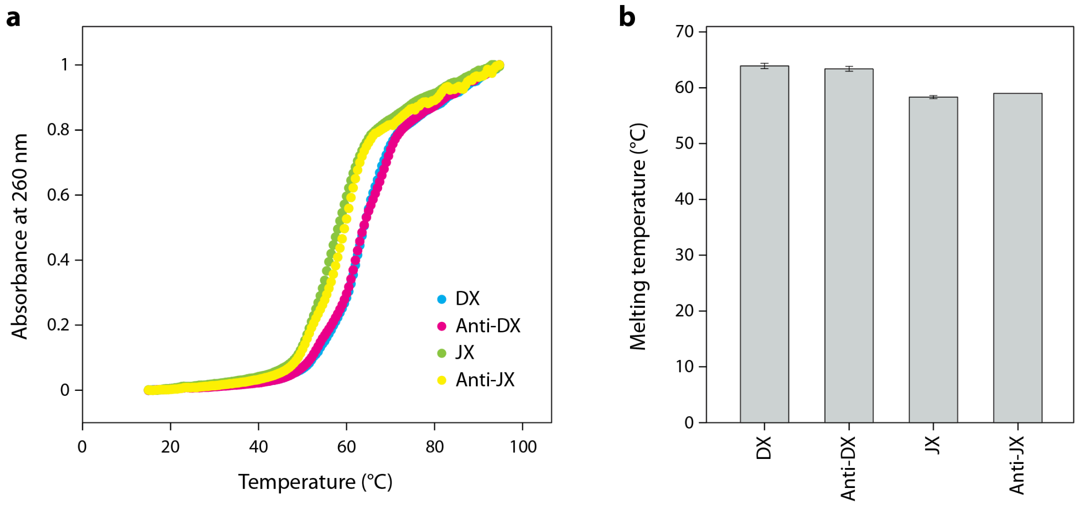

Figure S14. UV melting curves and melting temperatures for DX, JX and their corresponding anti-structures.
